# An Easily Integrable Industrial System for Gamma Spectroscopic Analysis and Traceability of Stones and Building Materials †

**DOI:** 10.3390/s21020352

**Published:** 2021-01-07

**Authors:** Marco Marini, Silvia Panicacci, Massimiliano Donati, Luca Fanucci, Erica Fanchini, Andrea Pepperosa, Massimo Morichi, Matteo Albéri, Enrico Chiarelli, Michele Montuschi, Kassandra Giulia Cristina Raptis, Andrea Serafini, Virginia Strati, Fabio Mantovani

**Affiliations:** 1 Department of Information Engineering, University of Pisa, via G. Caruso 16, 56122 Pisa, Italy; silvia.panicacci@phd.unipi.it (S.P.); massimiliano.donati@unipi.it (M.D.); luca.fanucci@unipi.it (L.F.); 2 CAEN S.p.A., via della Vetraia 11, 55049 Lucca, Italy; e.fanchini@caen.it (E.F.); a.pepperosa@caen.it (A.P.); m.morichi@caen.it (M.M.); 3 Department of Physics and Earth Sciences, University of Ferrara, via Savonarola 9, 44121 Ferrara, Italy; alberi@fe.infn.it (M.A.); chrnrc@unife.it (E.C.); montuschi@fe.infn.it (M.M.); rptksn@unife.it (K.G.C.R.); serafini@fe.infn.it (A.S.); strati@fe.infn.it (V.S.); mantovani@fe.infn.it (F.M.); 4 INFN, Legnaro National Laboratories, via dell’Università 2, 35020 Padua, Italy; 5 INFN, Ferrara Section, via Saragat 1, 44122 Ferrara, Italy

**Keywords:** gamma spectroscopy, industry 4.0, building material, NORM, industry, LoRa, WiFi, Cloud, euratom, RFID

## Abstract

In the building material and stones market, lots of restrictions are coming in different world zones. In Europe, a recent regulatory set up the maximum level of radiological emissions for materials intended for use in public and private building structures. For this reason, companies need to have a very efficient radiological measurements system in their production chain, in order to respect all the rules and to be competitive in the world market. This article describes CORSAIR, a Cloud-Oriented Measurement System for Radiological Investigation and Traceability of Stones. Our cyber-physical system consists of sensing nodes network connected to a data collection gateway through LoRaWAN protocol, and interfaces with a centralized cloud application. CORSAIR introduces a fast, repeatable, real-time and non-destructive method to measure radiological emissions and other parameters of each single building material item, uniquely identified by an applied RFID tag. The validity of this system is confirmed by in-situ measurement campaign compared with high-precision laboratory analysis. The results demonstrate the accuracy of the CORSAIR sensor and the possibility to easily integrate it in the company production chain without any change.

## 1. Introduction

The CORSAIR (Cloud-Oriented Radiation Sensor for Advanced Investigation of Rocks) was born to meet the recent acceptance of EU guidelines 2013/59/EURATOM [1] on safety standards for protection against ionizing radiations. The directive sets conditions for industrial sectors involving Naturally Occurring Radioactive Materials (NORMs): mainly oil and gas production, mining of ores, geothermal energy production and construction industry. For building materials such as alum-shale, granitoids (e.g., granites, syenite and orthogneiss), porphyries, tuff, pozzolana (i.e., pozzolanic ash) which are considered worrisome from a radiation protection point of view, several countries ask, before such materials are placed on the market, the activity concentrations of the natural radionuclides and effective dose are determined. In particular, the directive fixes at 1 mSv yr${}^{-1}$ the reference level of indoor external exposure to gamma radiation emitted by building materials. For practical monitoring purposes the threshold level is presented in the form of an Activity Concentration Index (*ACI*) that shall not exceed the value 1 (which correspond to 1 mSv yr${}^{-1}$). Before the EU directive there was no formal need to perform the radiological control based on gamma emission on each stone block or building materials. These measurements were bounded to research investigation, often connected with radon emanation and exhalation rate [2]. With the adoption of the EU Directive the radiological investigation of all the stone blocks has became mandatory, and new optimized measurement techniques are demanded by the stone market stakeholders.

In such a context, the developed CORSAIR equipment can be easily integrated in the traditional workflow of companies. It is designed to be managed by non-radiological experts, enabling operators to measure in real-time the level of natural radioactivity emission of the materials, in a non-destructive and time-efficient way, and to improve blocks’ traceability. The system is designed to represent a screening tool: only blocks exceeding the *ACI* threshold must be entrusted to a radiation protection expert assessing the possible restricting use of the material. The system introduces Industry 4.0 technologies and IoT applications in the stone market [3,4,5], increasing the competitiveness of the Italian supply chain with respect to developing countries (e.g., Brazil and China) which are currently experiencing a growth in the export numbers.

This work represents an extension of the paper Donati et al. [6] presented in the proceedings of the 2020 IEEE International Workshop on Metrology for Industry 4.0 and IoT. Hereafter, Section 2 compares CORSAIR with the state-of-the-art measurement methods. Section 3 describes the overall system architecture. Section 4 and Section 5 describe the cloud platform and the monitoring subsystem, respectively. Section 6 presents use-cases, procedures and performed tests. Section 7 describes some possible improvements to develop in the future versions of CORSAIR. Finally, conclusions are drawn in Section 8.

## 2. Comparison with the State-of-the-Art

Procedures and detection systems commonly used are totally different from the CORSAIR equipment (see Table 1). Many approaches require destructive tests in which the sample is reduced to powder, dried, poured into a container with a specific geometry and sealed. After a period of about 1 month required for establishing the radioactive equilibrium between ${}^{226}$Ra and ${}^{222}$Rn (see Figure 2 of [7]), the sample is positioned inside a thick shielding structure used to reduce the external background and measured with High Purity Germanium (HPGe) [8,9,10,11,12,13] or with a higher efficiency NaI spectrometer [13,14,15]. The analysis to extract the radionuclide abundances and the ACIs are done offline and manually. It requires often radiological experts and complexes procedures for calibrating the equipment. A portable and compact gamma spectrometer for ACI determination of building materials is described in [16]. Although it shows excellent performance, this proof of concept requires experts for measuring with specific geometry and data processing with offline software. For responding to EU Directive demands a dedicated, fast and user friendly detector is required.

The CORSAIR equipment uses a totally different approach in term of measurement type, measurement procedure and analysis, designing an automated system capable of providing a real-time non-destructive measurement of the *ACI*, natural radionuclide concentrations and effective dose for building materials according to the EU guidelines. In situ measurements are conducted through γ-ray spectroscopy on blocks of rock at quarries and processing centers, quantifying natural radionuclides in stone materials. Acquired data are synced through LoRa connectivity to a cloud database, where they can be easily accessed by sellers and buyers.

## 3. CORSAIR Architecture

**Figure 1:**
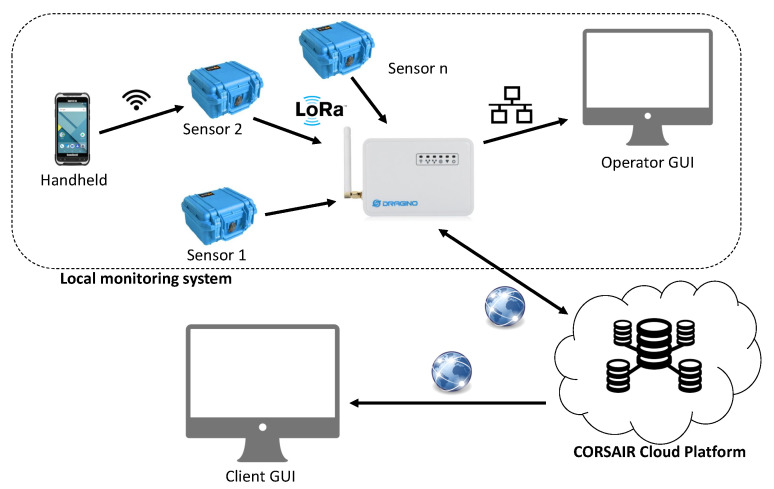
CORSAIR equipment architecture.

The overall CORSAIR equipment is composed by different subsystems interconnected in a start-of-stars network topology. The central element of the network is the Cloud Platform, which comprises storage and processing capabilities. It acts respectively as data collector with respect to peripheral subsystems and as data provider to the user. The distributed nodes of the network, namely the local monitoring systems, represent the equipment installed in the companies to allow to measure blocks and share data with the Cloud Platform. Such subsystems have an internal star topology. The overall architecture is shown in Figure 1.

The Cloud Platform is the main element of CORSAIR architecture. It has a twofold purpose. First of all, it offers online web-services for machine-to-machine (M2M) communications. In this way, a peripheral subsystem can upload or retrieve data to or from the Cloud Platform. Furthermore, it provides a web-based graphical user interface (GUI) thanks to a dedicated web-application that allows multi-users/profiles concurrent accesses for data consultation according to the SaaS (Software as a Service) paradigm. In order to accomplish its tasks, the Cloud Platform includes processing and storage capabilities. It runs in a common application server (e.g., Tomcat, etc.) and features and a relational database.

A generic CORSAIR local monitoring subsystem is organized as a sensors network, with a set of sensor nodes linked to a data gateway in a star topology. The data gateway exploits the Cloud Platform web-services for duplex data exchange. Additionally, it offers to the local user a dedicated web-based GUI to monitor the status of the entire sensors network. Indeed, each sensor node embeds a radiological measurement device with custom reading electronics besides all required processing, memory and wireless communication resources. The sensors node is powered by a rechargeable battery. A handheld device (i.e., rugged smartphone), with a dedicated Android application, enables the user to interact with the sensor node, providing a dedicated GUI to monitor and operate the equipment on the field. In such architecture, all sensing data measured by sensor nodes are gathered by the gateway and further pushed in the Cloud Platform, while periodical telemetry data generated by the nodes are made available locally on the data gateway.

The communication among all CORSAIR devices (handheld, sensor node, data gateway and cloud platform) is a critical part because they have very different capabilities and act in different scenarios. Handheld and sensor node communicate to each other through WiFi direct connections. This technology is preferable because such communications happen when the devices are close to each other, ensures an adequate data-rate, and it is also available or easily integrable on both sensor processing board and handheld. The LoRaWAN protocol [17] is used for real-time automatic communications of sensing and telemetry data from sensor nodes to the gateway, as seen in other sensing measurement scenarios [18,19,20,21]. This protocol is particularly suitable for these kinds of operational scenarios, ensuring a long distance of transmission without requiring the installation of infrastructural anchor nodes or repeaters. Moreover, data gateway uses Internet protocol (i.e., via LAN connectivity) to exchange data bidirectionally with the Cloud Platform. This cloud application allows the user to compare radiological data with regulatory thresholds established by the countries. This function is very useful for the customers and buildings materials companies.

An user profiling is implemented in the CORSAIR equipment. Each user can belong to one of three classes. A generic worker on the field has credential valid for logging in the handheld device to control and monitor sensor nodes as operator.

The company user class has the additional possibility to log in the local data gateway GUI for browsing and integrating information of all material items registered by the company. This class is suitable for all the employees.

Finally, for all the users interested in checking the characteristics of a material item after or before buying it, the client class is the most suitable. Users of this class are enabled to log in the Cloud Platform to retrieve all the information about the material. The only requirement is to know the item identification code.

## 4. Cloud Platform

The CORSAIR equipment has the Cloud Platform as a key component. Indeed, its task consists of collecting all data provided by companies’ data gateways, and to make them available to different kind of users and profiles, according to predetermined access policies. The Cloud Platform is currently deployed on a dedicated server provided by CAEN.

A generic data gateway uploads sensing data thanks to a set of REST web-services provided by the Cloud Platform, exploiting a pre-configured M2M credential for authentication purpose. On the platform, all incoming measured data are processed in order to calculate its radiological indexes and to compare such results with per-country *ACI* thresholds. Then all data are stored in the database for future consultations.

In addition to the radiological indexes, other material parameters are stored in the database. For instance, the ID that identify uniquely a material item (i.e., code of the RFID [22] applied on the item), its gamma spectrum, the timing and position (i.e., GPS coordinates) of the measurement, the company and operator who has performed the measurement. Additionally, a large set of optional data can be associated with the item: dimensions, colour, provenience, type of material, etc..

One of the most important features of the Cloud platform is the ability to present both raw and aggregated data, analysis results and traceability information for all registered material items. A dedicated web application provides these information upon user authentication. The authentication allows to identify user role and providing customized interface and personalised views of data.

**Figure 2:**
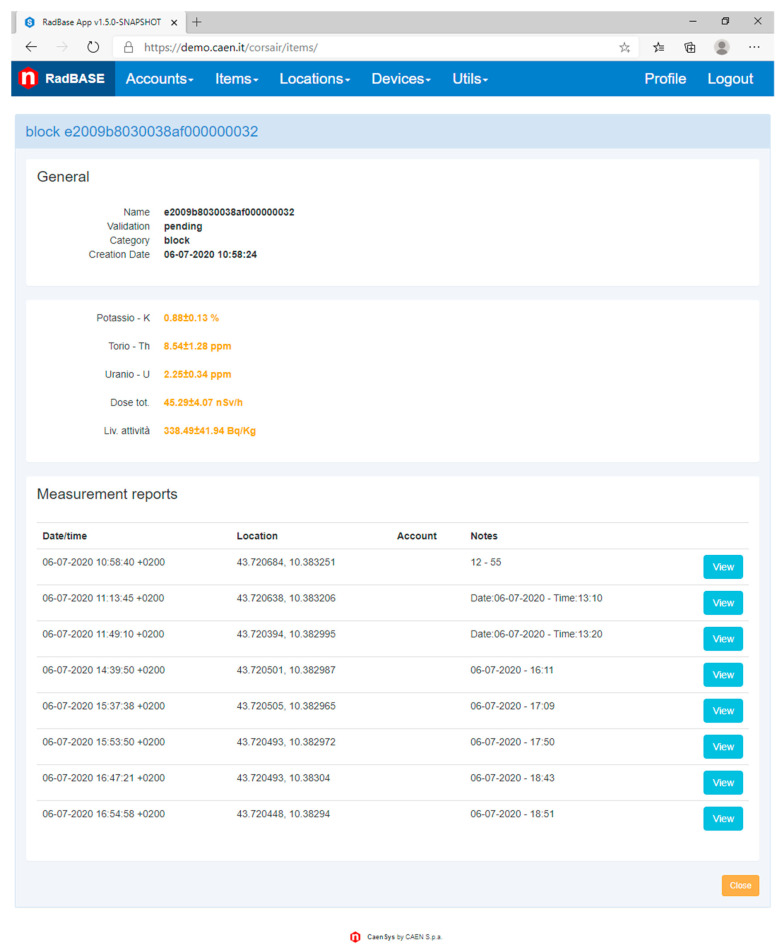
Cloud platform WEB page showing information about a block previously registered and measured.

For example, company users can browse the list of measured materials available in their warehouse, consult and integrate the information of each registered material. Instead, a generic client can control just the block that has bought or that wants to buy (the only requirement is the ID block). Figure 2 shows a cloud platform web page that contains all the information about a block previously measured.

## 5. Local Monitoring System

**Figure 3:**
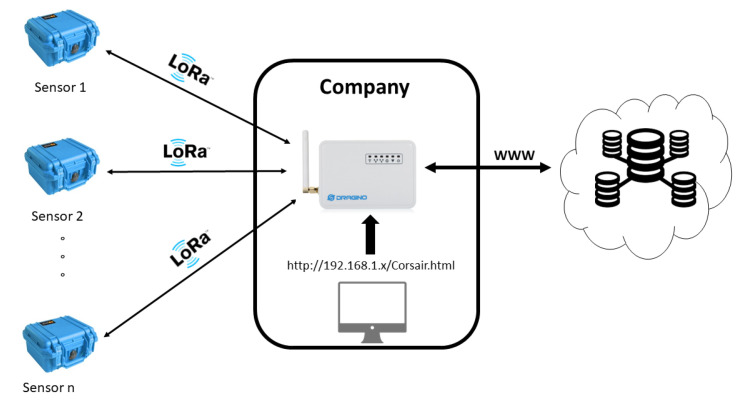
Local monitoring system architecture.

A company that wants to properly integrate the CORSAIR equipment into its operational chain, needs to use three different kinds of components: at least one sensor node, that measures the levels of different radionuclides and gamma spectra of materials under investigation; one data gateway that aggregate all sensing data coming from the sensor nodes and integrates them with additional physical and visual information; and finally at least one handheld device to interact with nodes (one handheld can control more than one node and one node can be controlled by more handhelds). All these components represent the local monitoring system. Figure 3 shows the architecture of the local monitoring system. The components are described in detail in the following subsections.

### 5.1. Sensor Node

The sensor node is the radiological sensor of the local monitoring system. It is a fully autonomous gamma spectroscopic device based on CAEN GammaStream [23]. The sensor includes a high-resolution scintillating detector coupled to a 14-pin photo-multiplier tube (PMT). This choice represents the optimal trade-off between energy resolution and a proper detection efficiency for the application purposes. A hollow cylindrical lead shield surrounding the detector permits to achieve a 60% reduction of the natural background. Thanks to the integrated Multi-Channel Analyzer (MCA) and to the onboard computational resources the device is able to acquire and process spectroscopic data in real-time. The main features of the sensor node are reported in Table 2.

The node can be operated and monitored via handheld device through WiFi direct connections (see Section 5.3). A specific thread running in the sensor node has the task of managing this communication channel. The sensor node implements a finite state machine with four main states (i.e., idle, measuring, recharge and error) and another thread controls its evolution based on signals received.

When it is started up, the sensor is in idle state which means that it is waiting for the *start* command form operator and it is monitoring the battery level. Once the start signal is received, the sensor goes to measuring state and starts the measuring procedure. It takes 30 min and during process the sensor collects information to send through the LoRa protocol at the end of the process. These information are: GPS coordinates, time and date, Operator ID, block RFID and natural radionuclides activities (see Section 6.1 for more details). If some error is rising (e.g., low battery or some internal errors), the sensor goes to error state until it receives a reset signal from the operator (see Section 5.3) and another LoRa packet is sent to update data gateway about the last error.

Furthermore, another thread is running in parallel on state machine. Its task is to send a LoRa packet periodically to the data gateway in order to update it about its status. Basically three different LoRa packets are generated by the sensor and each one is composed by a header that specifies the type and a payload that contains the information. The types of packet are:

- Measure packet: it is sent at the end of a measuring process and contains: sensor node ID, block RFID, operator ID, date, time, GPS coordinates and natural radionuclides activities.
- Update packet: it is periodic and contains information about the sensor node (sensor node ID, status, battery level, GPS coordinates, date, measuring time elapsed if it is measuring).
- Error packet: it is sent if an error is raised and it contains the sensor node ID and the error code.

**Figure 4:**
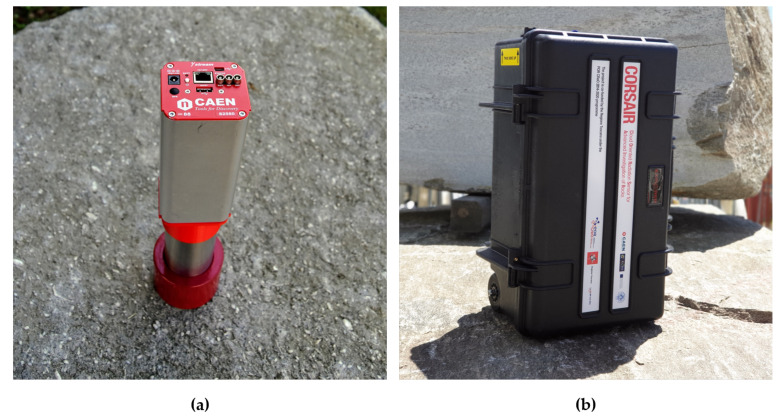
(**a**) Photo of the shielded CeBr${}_{3}$ detector coupled to the PMT and to CAEN GammaStream. (**b**) Photo of the CORSAIR sensor node enveloped by its shock-proof case.

Finally, the sensor is protected by a rugged case which takes care of its protection and of the heat dissipation (see Figure 4).

### 5.2. Data Gateway

The data gateway is the main component of the local monitoring system. It is based on a commercial programmable Dragino LoRaWAN gateway [24] and runs an ad-hoc developed software. It features a LoRaWAN interface to communicate with sensor nodes and a LAN connection to interact with the Cloud platform over Internet protocol. Furthermore, it has computational and tiny memory capabilities. From the functional point of view, it handles all data flows within, inwards and outwards the sensors network.

**Figure 5:**
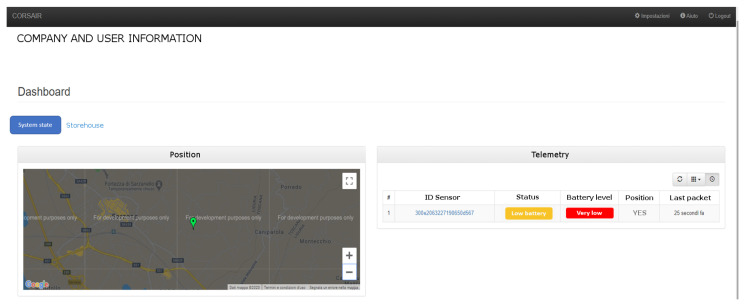
Web-based User Interface of data gateway to monitor the information coming from all company’s sensors.

The GUI offers to the user three main functionalities. Firstly, the monitoring of the sensor nodes information like status (idle, measuring or error), the measurement progression and the operator ID who has started it, GPS position, etc.. An example of the user interface is shown in Figure 5.

**Figure 6:**
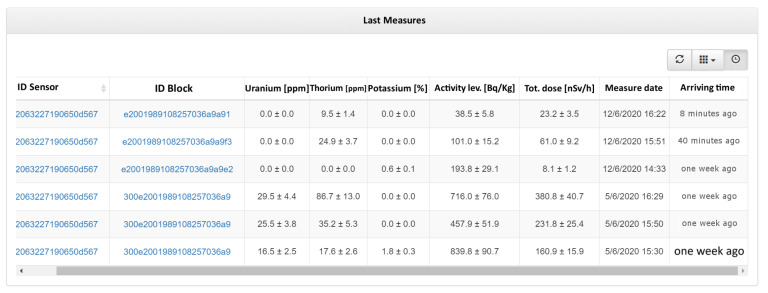
Web-based User Interface of data gateway showing measured material items owed by the company.

Secondly, it is also possible to retrieve and browse the data of all measured material items owned by the company, also requesting them to the Cloud Platform transparently if not available locally. Figure 6 shows the user interface when a user tries to see all measured material items.

**Figure 7:**
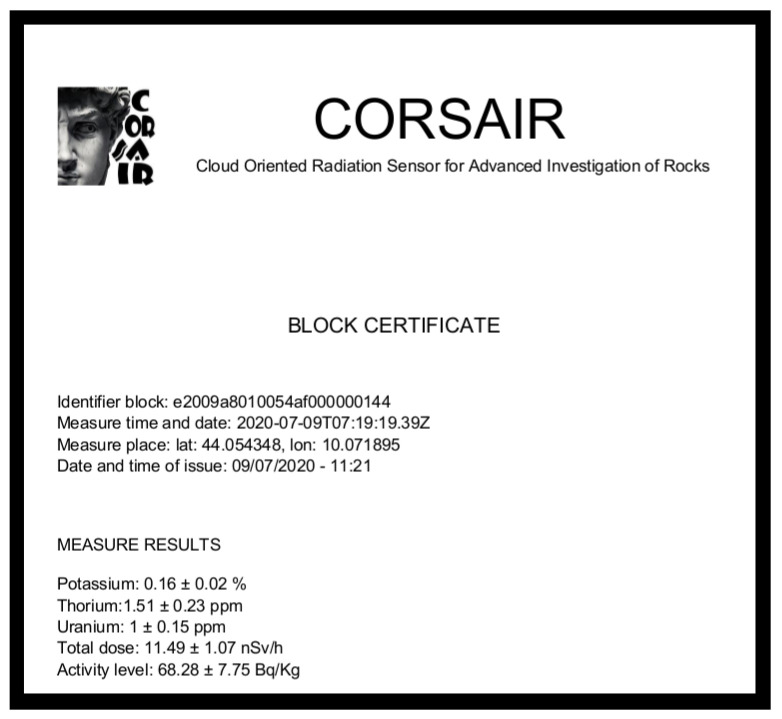
Material block certification generated by data gateway from company side.

Furthermore, for each measured material item it is possible to generate a certificate that contains all the information about the item like measurement location, date, timestamp and results. Of course, the information contained in the certification are also available in the Cloud Platform for authenticated users. An example of certification is shown in Figure 7.

Finally, the user interface allows to integrate the required/measured data concerning a material item with additional optional information. All the company employees who wants to access the gateway functionalities needs to enter user credentials through a dedicated web-based user interface.

### 5.3. Handheld Device

In order to interact with a sensor node, an operator should be equipped with a CORSAIR handheld device. In particular, it is an industrial, rugged smartphone equipped with a RFID reader, WiFi and 3G/4G connectivity. A dedicated Android software application (CORSAIR App), after a successful login, shows the operator ID, its company name and offers three different operations:

- Register a new material block in the cloud platform;
- Start a new measurement process;
- Connect to the sensor node in order to control it.

**Figure 8:**
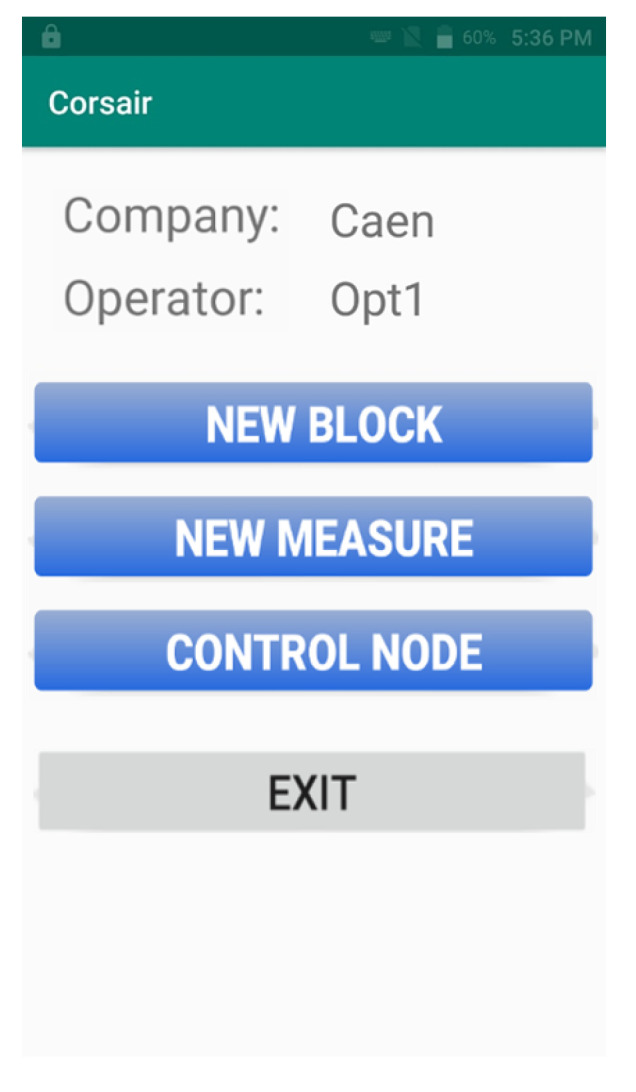
The main user interface of CORSAIR App.

Figure 8 shows the main user interface of CORSAIR App.

**Figure 9:**
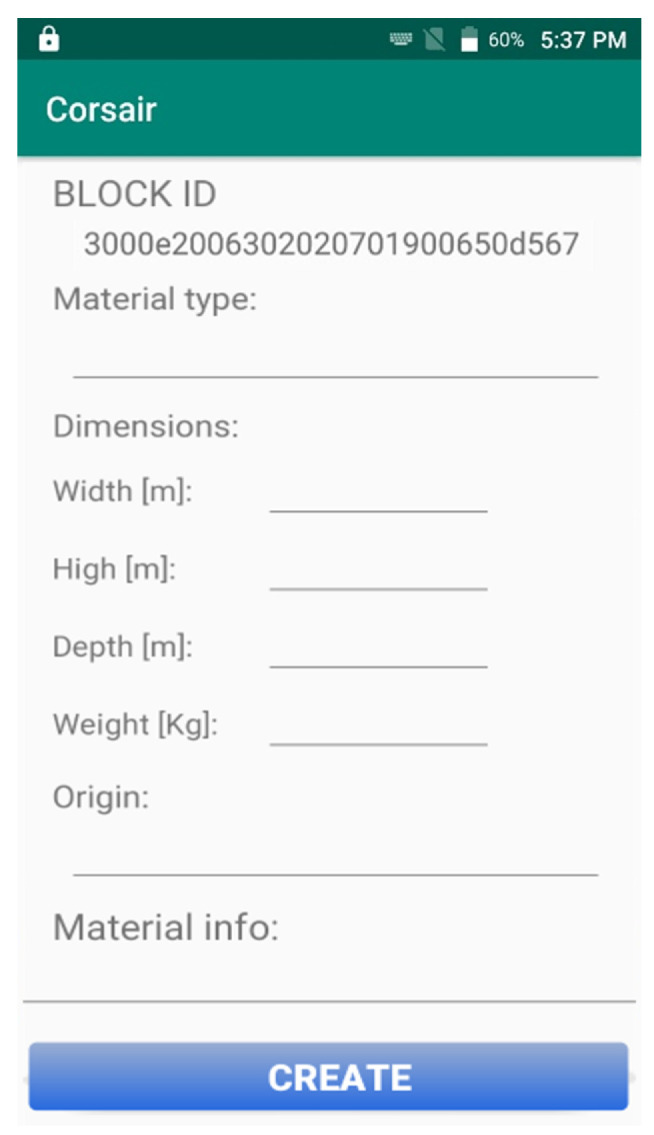
CORSAIR App fragment for the insertion of some information about a new block.

In case the user wants to register a new material block on the Cloud Platform, CORSAIR App leads the user in a step-by-step procedure. First, it is required to scan the RFID tag applied on the new material block. Then, more detailed information (photos, colour, dimension, etc) can be inserted thanks to the GUI. Figure 9 shows the CORSAIR App fragment asking to the user to add information about new material block. Finally, the Create button sends all the information to the Cloud Platform in order to initialize the new database item.

**Figure 10:**
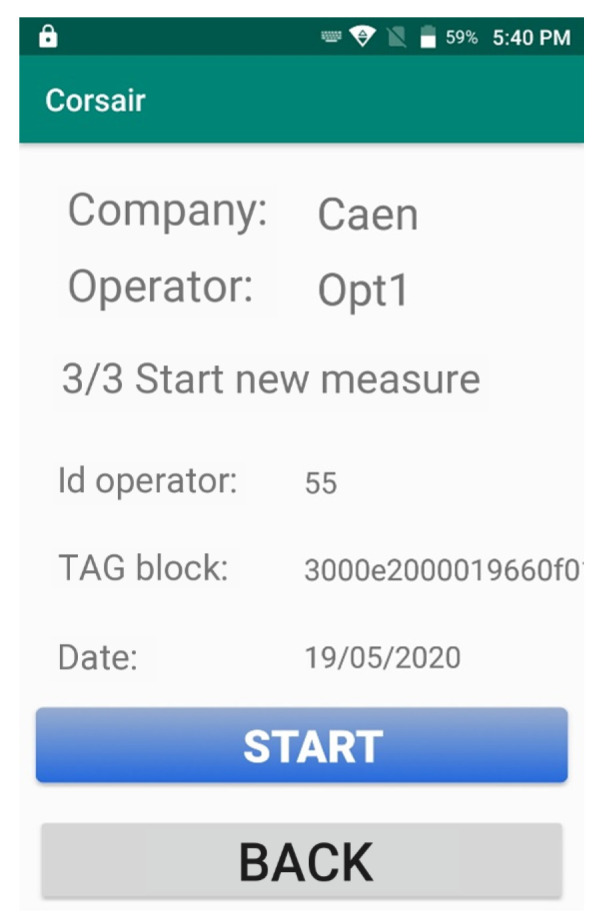
CORSAIR App screen after identification of sensor node and ID block, ready to start a new measurement procedure.

Also a guided procedure is available to start a new measurement. The CORSAIR App asks to the user to firstly identify the sensor node that will be used for the measurement and to identify the target block. Both operations are possible by scanning respectively the RFID tag of the sensor and the block with handheld device. Once identified the sensor and block, a new measurement can starts pressing the ’START’ button as shown in Figure 10. A more detailed description of measurement procedure is described in Section 6.1. The handheld provides notifications about the end of measurement processes.

**Figure 11:**
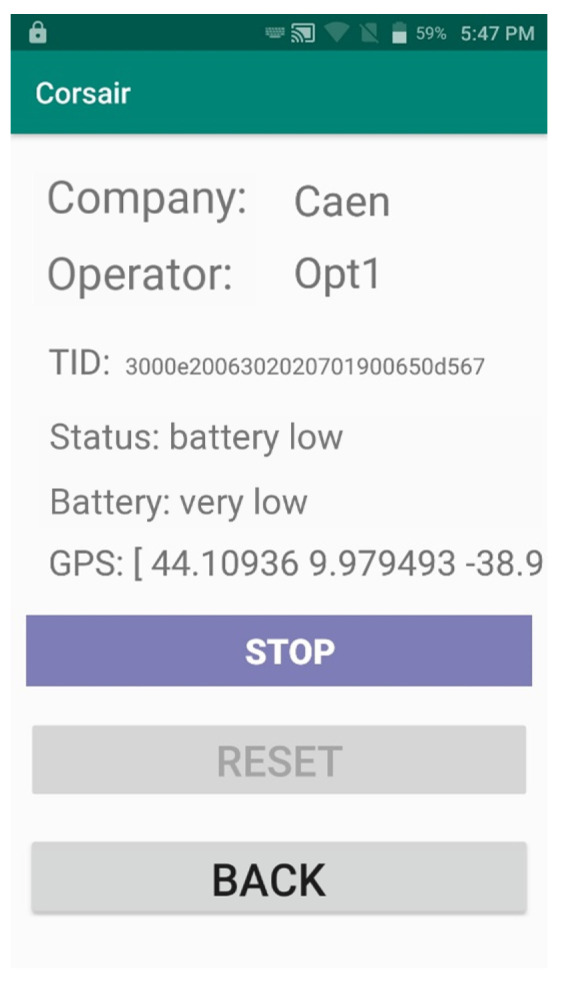
CORSAIR App screen that shows information about sensor node and allows the user to control it.

A direct connection between the handheld and the sensor node allows the user to control and monitor the sensor. In particular, the handheld connects via WiFi to a sensor node by scanning the internal RFID tag (i.e., network SSID is equal to RFID code). Once connected, the user can monitor the node (e.g., status, position, battery, errors, etc.), stop an ongoing measurement process, reset the node in case of errors and switch off the node. Figure 11 shows the CORSAIR App control sensor node screen.

## 6. Analysis Procedure and Experimental Test

The CORSAIR equipment foresees a demonstration of the system prototype in a real-life environment. The project consortium has organized a testing campaign in companies working in the stone market to validate the system and to demonstrate its applicability in the target sector.

If integrated in a company operational chain, the CORSAIR equipment makes it smarter and innovative adding a new procedure to measure gamma radiation emission of building materials in non-destructive way. Since it is conceived for non-radiological expert, it is easy to use and does not require any specific skill. All the information acquired from CORSAIR sensors are available among all stakeholders for commercial, traceability and regulatory purposes. It also reduces human errors and provides reliable and valuable information to both sellers and buyers.

### 6.1. Measurement Procedure

Once in the field, the operator places the trolley at the center top of the stone (usually consisting of a 3×3×2 m${}^{3}$ block). If not present, a RFID tag has to be applied to the stone surface. The tag permits the trace the block along the supply chain and the access to the available block information through the CORSAIR database. The operator logs in the CORSAIR App on the handheld device, scans the RFID tags on the stone and on the sensor node and starts a new measurement. The procedure allows the sensing data regarding the selected stone and the identity of the operator to be uniquely associated to the specific measuring node.

Once completed this part of the procedure, the software running on the sensor node begins its automatic 30 min measurement. The operator can monitor the progress of the measurement through the CORSAIR App or engage in other activities. During the process, the system continuously sends the status information and the remaining time to the gateway. A GPS position control algorithm integrated in the node ensures that the block and/or the sensor have not been moved during the entire measurement process. If the position is detected to change over a prefixed threshold, the control software interrupts the measuring process sending an error message to the gateway. Otherwise, the raw data and the geotagged analysis results are automatically sent to the data gateway and made available to company users through the Cloud Platform. In case of missing connection to the gateway, all data are temporary stored in the internal memory and synchronized once the connection is restored. A certification is also generated by data gateway at the end of measurement. This certificate can be printed out on-demand from the company and it can accompany the material block along its sales chain. The end of the process is notified through the handheld device to the operator, who can move the sensor node to another material block and proceed with a new measurement.

### 6.2. Analysis Procedure

**Figure 12:**
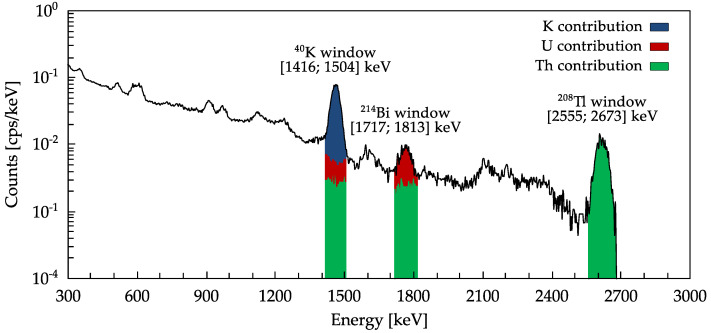
WAM procedure is applied to an experimental gamma spectrum measured by CORSAIR. The analysis is performed on the ${}^{40}$K, ${}^{214}$Bi and ${}^{208}$Tl energy windows, representative of the K, U and Th decay chains, respectively. For each window, the method enables the estimation of the contributions coming from the different decay chains, here depicted in different colours.

The gamma spectroscopic analysis is performed in real-time onboard of the sensor node. The spectral calibration reconstructing the energy of the incoming γ-photons from the MCA-digitized signals is performed through a stochastic minimization algorithm based on simulated annealing [25]. The result is a calibrated gamma spectrum (see Figure 12), that is an histogram of the recorded gamma events (expressed in counts per second, *cps*) as a function of the photon energy (expressed in keV). The identity and quantity of the gamma emitters present in the block is assessed through the analysis of the so-called photopeaks, the gaussian-shaped structures identifiable in the spectrum. The conventional approach requires the analysis of the energy windows around the photopeaks of ${}^{40}$K (1460 keV), of ${}^{214}$Bi (1765 keV) and of ${}^{208}$Tl (2614 keV) through the so called Windows Analysis Method (WAM) [10,26], graphically depicted in Figure 12. Under the assumption of secular equilibrium this method permits the assessment of the specific activity of potassium $C_{K}$ and to indirectly estimate the specific activities of the radionuclides belonging to the U and Th decay chains respectively (hence referred to as equivalent $C_{U}$ and $C_{Th}$, namely $C_{eU}$ and $C_{eTh}$).

The use of WAM requires a careful experimental calibration in order to disentangle the different contributes due to K, U and Th. Standard calibration techniques [26] do not take into account the presence of background contamination and cannot be straightforwardly applied to new generations detectors (e.g., CeBr${}_{3}$ scintillators). Hence, the sensor node and the rock blocks of interest have been recreated in a semi-infinite planar environment in GEANT4 [27]. The simulations of the veracious experimental setup and measuring parameters permitted to properly calibrate the sensor node and to optimize the shielding thickness as a function of the signal to noise ratio. The quality of the calibration and of the Monte Carlo simulation has been experimentally validated a posteriori (Section 6.3).

Starting from the specific activities $C_{K}$, $C_{eU}$ and $C_{eTh}$ resulting from the automatic WAM algorithm, the effective dose *D* is evaluated by the onboard computing unit as:

(1) $$D\left[nSv\;h^{-1}\right]=0.03\frac{nSv\;h^{-1}}{Bq\;kg^{-1}}\cdot C_{K}+0.31\frac{nSv\;h^{-1}}{Bq\;kg^{-1}}\cdot C_{eU}+0.42\frac{nSv\;h^{-1}}{Bq\;kg^{-1}}\cdot C_{e}Th$$

where the specific activities $C_{K}$, $C_{eU}$ and $C_{eTh}$ expressed in $Bq\;kg^{-1}$ are properly weighted by using the respective dose coefficients [28]. In the same way, the *ACI* is calculated from the measured specific activities as:

(2) $$ACI=\frac{C_{eU}}{300\;Bq\;kg^{-1}}+\frac{C_{eTh}}{200\;Bq\;kg^{-1}}+\frac{C_{K}}{3000\;Bq\;kg^{-1}}$$

Once finished the analysis process, the sensor node informs the operator and readily sends the acquired spectrum, the measured specific activities $C_{K}$, $C_{eU}$ and $C_{eTh}$ as well as the resulting effective dose *D* and the *ACI* (CORSAIR makes available a list of radiological indexes, in addition to the European ACI equation [2], related to more than 20 international markets) to the data gateway.

### 6.3. *In-Situ* Experimental Tests

The CORSAIR equipment underwent a first laboratory examination aimed at verifying the functioning of the equipment, the wireless data transfer protocol as well as to test the correct integration of the analysis software running on the single-board embedded computer onboard of the sensor node. Hence, it was organized an in-situ measurement campaign, aimed at validating the Monte Carlo simulation and the correct calibration of the detector.

**Figure 13:**
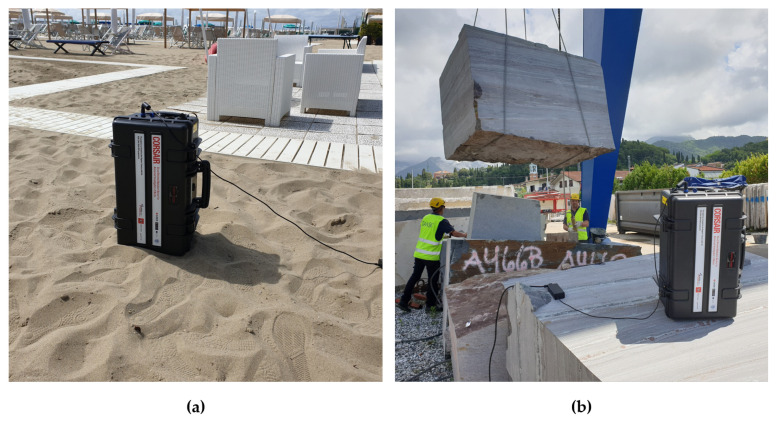
(**a**) Photo of the CORSAIR equipment during the M1 measurement on sand. (**b**) Photo of the equipment performing the M2 measurement on a typical 3×3×2 m${}^{3}$ granite block.

A first test M1, performed on the sand (chosen because of its homogeneity) permitted to compare the experimental measurement to the Monte Carlo simulated semi-infinite planar environment used for the calibration. Then, the CORSAIR equipment was tested in its use case scenario at a processing center to verify its performances and accuracy on a series of typical blocks. The measurement M2, performed on a block of granite, is here reported as a reference. Figure 13 shows the prototype in operation during the M1 and M2 measurements.

The performance of the CORSAIR detector has hence been compared to those of MCA_Rad [29], a high-resolution apparatus used for careful laboratory radiometric analysis. The MCA_Rad system is made up two coaxial p-type HPGe detectors having an energy resolution of approximately 0.22% at ${}^{137}$Cs, surrounded by 10 cm of lead and 10 cm of copper on each side to reduce the environmental background. Analysis has been performed on two 180 cm${}^{3}$-volume samples of sand and granite, collected during the measurement campaign by gathering the sand underneath the detector during M1 and by removing a part of the granite block measured in M2. The comparison between the CORSAIR and the MCA_Rad measured radiological information is reported in Table 3.

The CORSAIR equipment estimated effective dose *D* and *ACI* shows an excellent agreement with the laboratory analysis for both M1 and M2. All the quantities measured in-situ by the prototype are compatible within 1σ with the MCA_Rad results with the exception of M1’s measured $C_{K}$ (which ends up not affecting the dose and ACI assessment). The CORSAIR equipment proves to represent a reliable fully autonomous screening tool, able to provide accurate radiological measurement to non-expert users.

## 7. Future Works

The actual CORSAIR equipment offers all the functionalities that a company needs to compete at the best in the building material and stones market. Despite this, we are working on adding more functionalities to improve our system in terms of robustness and usability. CORSAIR equipment may face a multi-sites scenario or has to cover a wide area by its sensors network. For this reason, embedding a meshed LoRaWAN network infrastructure [30] in CORSAIR makes it suitable also for these specific scenarios. We are also planning to add 4G connection on sensor node to make it more reliable. Indeed, in case of LoRa device failure, the sensor could not communicate to the gateway anymore. Furthermore, 4G also allows the sensor to work directly on extraction sites, where LoRa gateway setup could be difficult and Internet cable missing. Finally, a navigation function could be useful for very huge companies. This functionality leads the operator to a sensor node that has finished a measurement. It could be possible to exploit sensor GPS coordinates.

## 8. Conclusions

This article describes the CORSAIR equipment, a system designed to help companies that work in building materials and stone markets, to be compliant with the EU directive in term of radiological characterization of the stone blocks.

The CORSAIR equipment exploits several cutting edge technologies like LoRa, WiFi and 4G as communication protocol, GPS coordinates, RFID tags, SaaS cloud platform, gamma spectroscopic analysis and radiological sensors. All these components cooperate together for non-destructive measurement, information sharing of stones (accessible with a very simple web-based user interface) and improve the traceability of blocks by exploiting GPS.

A comparison between in situ surveys and in laboratory high-precision measurements has shown an excellent agreement: the specific activities, effective doses and ACI are compatible within 1σ. These promising results require to be validated with higher statistics, with different commercial rocks, in various operative situations. Since the scalability and integrability of CORSAIR need to be tested in the operational production chain, the building materials companies are encouraged to include it in their equipment pools.

## Figures and Tables

**Table 1:** Essential review of the main features of gamma spectrometers dedicated to radiological characterization of building material.

	HPGe [8,9,10,11,12,13]	NaI [13,14,15]	B-NORM (LaBr${}_{3}$) [16]	CORSAIR (CeBr${}_{3}$)
Not destructive meas.	No	Yes	Yes	Yes
Back-end time	≥21	≥21	0	0
Energy Res. (@ 662 keV)	∼0.3%	∼7%	∼3%	∼3.8%
Measurement time	day(s)	hours	n.a.(∼hours)	30 min
Sample preparation	Yes	Yes	No	No
Background meas.	Yes	Yes	Yes	No
Automatic data analysis	No	No	No	Yes
Automatic ACI calculation	No	No	No	Yes
Simulation of the experimental set-up	by construction	by construction	required	defined by procedure
Expert personnel for using the equipment	Yes	Yes	Yes	No
Shielding	Yes	Yes	No	Yes
On-site measurement	No	No	Yes	Yes
Stable experimental setup	No	No	Yes	No
Wireless data transfer	No	No	No	Yes
Sensors network cloud/IoT based	No	No	No	Yes

**Table 2:** List of the main features of CORSAIR sensor node detector and computational unit.

Sensor Node Features	
Detection unit	2″×2″ cylindrical CeBr${}_{3}$
Energy resolution	3.8% at ${}^{137}Cs$
Background reduction	1.25cm-thick lead shield
Envelope	IP67 shock-proof trolley
Positioning system	Embedded GPS receiver
Communication capabilities	Ethernet, USB 2.0, Bluetooth^®^, Wi-Fi, LoRa
Computational unit	Industrial Beaglebone Black
Storage	2 GB internal SSD memory
Onboard operating system	Linux^®^ OS
Battery	13.4 mAh
Weight	less than 16 kg

**Table 3:** Comparison between the specific activities, effective doses and ACI estimated by the CORSAIR equipment during the M1 and M2 measurements and the corresponding laboratory analyses performed by the MCA_Rad apparatus.

ID	Detector	C_K_ [Bq kg^−1^]	C_eU_ [Bq kg^−1^]	C_eTh_ [Bq kg^−1^]	D [nSv h^−1^]	ACI
M1	CORSAIR	556±83	32±5	51±8	47.4±4.2	0.55±0.05
	MCA_Rad	889±53	27±2	41±3	51.6±2.2	0.59±0.03
M2	CORSAIR	454±68	20±3	22±3	28.8±2.6	0.33±0.03
	MCA_Rad	498±31	17±11	20±2	28.3±3.8	0.32±0.04

## References

[1] (2013). Council Directive 2013/59/EURATOM Laying Down Basic Safety Standards for Protection against the Dangers Arising from Exposure to Ionising Radiation, and Repealing Directives 89/618/Euratom, 90/641/Euratom, 96/29/Euratom, 97/43/Euratom and 2003/122/Euratom.

[2] Schroeyers W. (2017). Naturally Occurring Radioactive Materials in Construction: Integrating Radiation Protection in Reuse.

[3] (2014). Indagine Congiunturale Sul Settore Lapideo Italiano 2014.

[4] (2016). Confindustria Verona, "Il Settore Lapideo in Italia e nei due Maggiori Distretti: L’Apuo-Versiliese e il Distretto Veneto".

[5] (2000). L. Maraviglia "Terzo Rapporto di Ricerca: I Distretti Lapidei e le Sfide Della Globalizzazione", Progetto Equal Fase II.

[6] Donati M., Marini M., Fanucci L., Fanchini E., Morichi M. A Cloud-Oriented Measurement System for Radiological Investigation and Traceability of Stones. Proceedings of the 2020 IEEE International Workshop on Metrology for Industry 4.0 & IoT.

[7] Xhixha G., Alberi M., Baldoncini M., Bode K., Bylyku E., Cfarku F., Callegari I., Hasani F., Landsberger S., Mantovani F. (2016). Xhixha Kaceli, Calibration of HPGe detectors using certified reference materials of natural origin. J. Radioanal. Nucl. Chem..

[8] Ahmad N., Nasir T., Rafique M., Rizwan S. (2019). Natural radioactivity and associated radiological hazards in limestone used as raw material in cement of Lucky Cement Factory, Pezu, Pakistan. Int. J. Environ. Anal. Chem..

[9] Verde G.L., Raulo A., D’Avino V., Roca V., Pugliese M. (2020). Radioactivity content in natural stones used as building materials in Puglia region analysed by high resolution gamma-ray spectroscopy: Preliminary results. Constr. Build. Mater..

[10] Caciolli A., Baldoncini M., Bezzon G.P., Broggini C., Buso G.P., Callegari I., Colonna T., Fiorentini G., Guastaldi E., Mantovani F. (2012). A new FSA approach for in situ gamma ray spectroscopy. Sci. Total Environ..

[11] Sanjuán M.Á., Suárez-Navarro J.A., Argiz C., Mora P. (2020). Assessment of natural radioactivity and radiation hazards owing to coal fly ash and natural pozzolan Portland cements. J. Radioanal. Nucl. Chem..

[12] Turhan Ş., Arıkan İ.H., Demirel H., Güngör N. (2011). Radiometric analysis of raw materials and end products in the Turkish ceramics industry. Radiat. Phys. Chem..

[13] Khan I.U., Sun W., Lewis E. (2019). Review of low-level background radioactivity studies conducted from 2000 to date in people Republic of China. J. Radiat. Res. Appl. Sci..

[14] Celik I.C., Mehmet K. (2019). Assessment of Environmental Radioactivity and Health Hazard in Soil, Water, and Stone Samples in Siverek Town of Sanliurfa Province in Southeastern Turkey. Procedia Comput. Sci..

[15] Dentoni V., Pelo S.D., Aghdam M.M., Randaccio P., Loi A., Careddu N., Bernardini A. (2020). Natural radioactivity and radon exhalation rate of Sardinian dimension stones. Constr. Build. Mater..

[16] Stals M., Verhoeven S., Bruggeman M., Pellens V., Schroeyers W., Schreurs S. (2014). The use of portable equipment for the activity concentration index determination of building materials: Method validation and survey of building materials on the Belgian market. J. Environ. Radioact..

[17] Martin B., Vidler J.E., Roedig U. (2016). LoRa for the Internet of Things.

[18] Camboim M.M., Oliveira V.S., Villarim M.R., Villarim A.W.R., Catunda S.Y.C., Baiocchi O., De Souza C.P. (2020). An Online Remote Verification System of Thermal Sources for Energy Harvesting Application. IEEE Trans. Instrum. Meas..

[19] Rizzi M., Ferrari P., Flammini A., Sisinni E. (2017). Evaluation of the IoT LoRaWAN Solution for Distributed Measurement Applications. IEEE Trans. Instrum. Meas..

[20] Yi-Kang K., Seung-Yeon K. (2020). Success Probability Characterization of Long-Range in Low-Power Wide Area Networks. Sensors.

[21] Kufakunesu R., Hancke G.P., Abu-Mahfouz A.M. (2020). A Survey on Adaptive Data Rate Optimization in LoRaWAN: Recent Solutions and Major Challenges. Sensors.

[22] Want R. (2006). An introduction to RFID technology. IEEE Pervasive Comput..

[23] CAEN SpA GAMMASTREAM–Digital MCA Tube Base for Gamma-Ray Spectroscopy. https://www.caen.it/products/gamma-stream/.

[24] Dragino LoRaWAN Gateway. https://www.dragino.com/products/lora-lorawan-gateway.html.

[25] Hwang C. (1988). Simulated Annealing: Theory and Applications.

[26] Erdi-Krausz G., Matolin M., Minty B., Nicolet J.P., Reford W.S., Schetselaar E.M. (2003). Guidelines for Radioelement Mapping using Gamma Ray Spectrometry Data.

[27] Agostinelli S., GEANT4 Collaboration (2003). GEANT4–a simulation toolkit. Nucl. Instrum. Meth. A.

[28] (2010). United Nations Scientific Committee on the Effects of Atomic Radiation, “UNSCEAR 2008 Report: Sources and Effects of Ionizing Radiation”.

[29] Xhixha G., Bezzon G.P., Broggini C., Buso G.P., Caciolli A., Callegari I., Shyti M. (2013). The worldwide NORM production and a fully automated gamma-ray spectrometer for their characterization. J. Radioanal. Nucl. Chem..

[30] Lee H., Ke K. (2018). Monitoring of Large-Area IoT Sensors Using a LoRa Wireless Mesh Network System: Design and Evaluation. IEEE Trans. Instrum. Meas..

